# A Detailed Phylogenetic Analysis of FIV in the United States

**DOI:** 10.1371/journal.pone.0012004

**Published:** 2010-08-09

**Authors:** Eric A. Weaver

**Affiliations:** Division of Infectious Diseases, Department of Internal Medicine, Mayo Clinic, Rochester, Minnesota, United States of America; Institute of Infectious Disease and Molecular Medicine, South Africa

## Abstract

**Background:**

Feline immunodeficiency virus (FIV) is a lentivirus associated with AIDS-like illnesses in cats and has been used as a model for the study of human immunodeficiency virus (HIV). A feature of HIV and FIV infection is the continually increasing divergence among viral isolates between different individuals, as well as within the same individuals.

**Methodology/Principal Findings:**

The goal of this study was to determine the phylogenetic patterns of viral isolates obtained within the United States (U.S.) by focusing on the variable, V3-V4, region of the FIV envelope gene.

**Conclusions/Significance:**

Data indicate that FIV, from within the U.S., localize to four viral clades, A, B, C, and F. Also shown is the geographic isolation of strains where clade A and clade B are found predominately on the west coast; however, clade B is also found throughout the U.S. and represents the predominant clade. This study presents a complete and conclusive analysis of FIV isolates from within the U.S. and may be used as the essential basis for the development of an effective multi-clade vaccine.

## Introduction

Feline immunodeficiency virus was first isolated from a cat exhibiting an immunodeficiency-like syndrome [1]. This isolate from petaluma, california was designated the petaluma strain, and has become one of the most common laboratory strains used for research investigating lentiviral pathogenesis, antiviral chemotherapy design and testing, and for developing vaccine strategies as a small animal model for HIV [2], [3], [4], [5], [6], [7]. Further investigation lead to the discovery that FIV had a wide range of host species and was prevalent in animals at a rate of 1% to 14%, depending on the country, age, gender, and risk of exposure [1], [8], [9], [10], [11], [12], [13]. Sick animals are two to three times more likely to be infected with FIV than clinically healthy cats [9].

FIV, like the primate lentiviruses, readily infect CD4^+^ T [14]. However, fiv has been found to infect and replicate in a wide range of host cells including: CD8^+^ T lymphocytes, macrophages, astroglial cells, and kidney cells [12],[15],[16],[17]. Although not fully understood, the receptor for FIV is believed to be CD9 [18]; whereas, SIV and HIV use the CD4 receptor as well as the coreceptors CCR5 and CXCR4 for entry [19], [20]. FIV results in a disease progression similar to HIV and SIV and is associated with symptoms of immunodeficiency: weight loss, chronic lesions, opportunistic infections, and neurological abnormalities [21]. Due to the similarities in FIV and HIV pathogenesis, fiv has become as the only small animal non-primate model for the study of HIV disease, therapy, and prophylaxis.

As in HIV, FIV has a large amount of genetic variation and can be grouped into clades based on the nucleotide sequence and geographic location [22],[23]. This vast amount of genetic variation has lead to an almost insurmountable impediment [24]. Studies on FIV in countries, such as Japan, Italy, and Brazil have been shown to consist primarily of one clade of endogenous FIV. However, FIV in Australia, New Zealand, Canada, and Japan has been shown to consist of multiple clades [22], [23], [24], [25], [26], [27], [28], [29], [30], [31]. This large amount of genetic variation in the u. S. Is also complicated by the existence of recombinant viral strains [32], [33].

Recently, a dual clade vaccine (Fort Dodge City) was advanced into commercial production. This vaccine is composed of two distinct clades, A and D [34]. As the amount of genetic variation in the viral challenge will be inversely proportional to the efficacy of the vaccine, it was of interest to examine the phylogenetic variation of FIV within the United States.

Thirty-six FIV isolates were obtained from infected domestic and feral cats in eight U.S. cities. The proviral DNA was used to amplify the V3-V4 envelope gene for phylogenetic analyses as compared to other known U.S. isolates. The phylogenetic analyses indicate that the U.S. is populated with a much greater divergence of FIV than previously thought. Data indicate high levels of divergence across the country and within any one city. The resulting mixture of viral clades has, as expected, resulted in the continuous evolution of recombinant virus strains.

## Materials and Methods

### Cells and DNA isolation

FIV *in vivo* infection within PBMCs was confirmed by antibody ELISA. Whole blood was collected in EDTA (K_3_) tubes and shipped overnight. Samples from domestic and feral cats that tested positive for FIV by *in vitro* assay were voluntarily submitted from the following hospitals: Stanford University Feral Cat Association, Stanford, CA, Dunstable Animal Clinic, Dunstable, MA, Oregon Feral Cat Coalition, Portland, OR, Homer Veterinary Clinic, Homer, AK, Betts Sanderson, San Ramon, CA, Tree House Animals, Chicago, IL, and Brazos Feral Cat Association, College Station, TX. PBMCs were processed using a BSL2 isolation cabinet. Lymphocytes were isolated from whole blood using a 5-fold volume of ACK lysis buffer (0.15 M NH_4_Cl, 10 mM KHCO_3_, 0.1 mM EDTA, pH 7.4) and washed 1 time with PBS. Genomic DNA (gDNA) that contained the FIV proviral DNA was isolated from the lymphocytes using a mammalian genomic DNA Isolation miniprep kit (Sigma, St. Louis, MO). The gDNA was stored at –20°C until it was amplified by PCR.

### PCR

gDNA containing integrated proviral FIV DNA isolated from lymphocytes was used as the template for PCR. Briefly, the envelope V3-V4 genes were amplified by nested PCR as previously described [35]. The primary amplification of the V3-V4 envelope region was performed using the primers 6785F (5′-GCGCAAGTAGTGTGGAG-3′) and 8842R (5′- GCTTCATCATTCCTCCTCTT-3′). PCRs were performed in a total volume of 50 ul containing 100 ng gDNA, 100 nM final concentration of each primer, 200 uM of each of the four dNTPs, and 1.5 U Taq DNA polymerase. The following parameters were used to amplify the genes using a GeneAmp 2400 (Perkin Elmer): 94°C for 5 min. followed by 5 cycles of 94°C for 60 s, 53°C for 60 s, and 72°C for 2 min., and finally 30 cycles of 94°C for 15 s, 53°C for 45 s, and 72C for 2 min. The annealing step was increased by 0.1°C per cycle for 30 cycles. The reaction was held at 72°C for 15 min. followed by 4°C. The following primers were used to amplify the V3–V4 region of the envelope in a secondary PCR reaction: 7316F (5′-ATACCAAAATGTGGATGGTG-3′) and 7866R (5′-CAAGACCAATTTCCAGCAAT-3′). The secondary PCR mixture was identical to the primary PCR with the exception that 5 ul of the primary PCR was used as the template. The thermal cycling parameters for the secondary PCR was identical to the primary PCR with the exception that the extension step was shortened to 72°C for 1 min. The annealing step was increased by 0.1°C per cycle for the 30 cycles. The reaction was held at 72°C for 7 min. followed by 4°C. DNA isolated from FIV negative lymphocytes served as negative controls.

### DNA sequencing

The PCR products were purified using a Genelute PCR Clean-up kit (Sigma, St. Louis, MO). The PCR products were adjusted to 5 ng/ul and 2 ul was used for direct sequencing. DNA sequencing was performed using BigDye Terminator v3.1. The following thermal cycling steps were used for extension of the V3–V4 product for 45 cycles: 96°C for 10 sec, 50°C for 5 sec and 60°C for 4 min. After purification of the product by ethanol precipitation or spin-column purification protocol, the samples were sent to the Gene Technologies Core Facility for DNA sequencing and analyzed using the ABI 3100 Automated Sequencer (Department of Biology, Texas A&M University). The products were sequenced in triplicate using the forward and reverse PCR primers. A consensus sequence was determined by aligning the six sequences in the Sequencher program.

### Sequence alignment and phylogenetic analyses

Sequences included in the phylogenetic analysis were obtained from this study and from previous GenBank entries (Table S1). Sequences were aligned by using the Clustal X program [36]. All aligned sequences were then inspected manually to correct for apparent mistakes. Positions containing gaps or ambiguously aligned positions were removed from the datasets. Phylogenetic trees were created using the program PAUP* 4.0b8 (Sinauer Associates, Sunderland Mass.) and TreePuzzle 5.0 (Free Software Foundation, Inc. Boston, MA). Maximum parsimony analyses are performed in order to establish evolutionary relationships based on the least number of steps to explain the tree. The following parameters were used for maximum parsimony of envelope V3–V4 nucleotide sequences: i) 572 total characters, ii) all characters were weighted equally, iii) 215 characters were constant, iv) 277 parsimony informative characters, v) random addition of sequence, vi) 500 bootstrap replicates. Maximum likelihood analyses attempt to infer the evolutionary tree that has the highest probability of observing the data. Maximum likelihood analysis continually compares trees and chooses the one with the best score. The following parameters were used for maximum likelihood puzzling tree based on nucleotide sequences: i) 575 total characters, ii) 163 constant characters, iii) 395 site patterns, iv) HKY model of substitution, v) expected transition/transversion ratio  = 1.75, vi) pyrimidine transition/purine transition ratio  = 0.41, vii) 1215450 quartets were analyzed with 17686 unresolved quartets, viii) 100 puzzling steps were used for statistical analysis. Distance analyses compare pairwise characters in a data set column and determine the degree of differences. Distance trees of the nucleotide sequence data were established using the following parameters: i) 572 characters, ii) distance measure  =  LogDet/paralinear, iii) ties were broken at random, iv) 1000 bootstrap replicates were used for statistical analysis.

The maximum likelihood phylogenetic analysis of the amino acid sequence was established using the following parameters: i) 290 characters, ii) 229 site patterns, iii) 13 constant site patterns, iv) model of substitution  =  JTT, v) 1663740 quartets were analysed with 146464 unresolved quartets, vi) 1000 puzzling steps were used for statistical analysis. The following parameters were used in creating the maximum parsimony tree of the amino acid alignment: i) 298 characters, ii) all characters have equal weight, iii) 109 constant characters with 130 parsimony-informative characters, iv) gaps were treated as missing, v) 500 bootstrap replicates with full heurisistic search was used for statistical analysis. The following parameters were used to establish the distance phylogenetic tree based on the amino acid sequence alignment: i) 298 characters, ii) bootstrap method with neighbor-joining search at 1000 replicates was used for statistical analysis, iii) ties were broken at random, iv) distance measure  =  mean character difference. All trees were viewed in treev32 (Roderick M. Page, 2001).

## Results

In order to define the level of FIV genetic diversity in the U. S., an epidemiological survey of FIV envelope sequence diversity was conducted. Blood specimens were obtained from FIV infected cats as a result of requests sent to Veterinarians and researchers. Genomic DNA was isolated and nested PCR was used to amplify a predicted 550 bp segment of the envelope gene that encodes the viral surface protein. This fragment spans the third and fourth variable regions. The PCR products ranged in size from 443 bp to 555 bp. The average size of the isolates, which clustered to the A, B, and F branches were 501.0 bp ±10.34, 495.5±10.60, and 521.4±8.02, respectively. Table S2 shows the sample descriptions and clade analysis as obtained from the current study as well as from previously published studies [24], [31]. Many of the sequences were classified using a heteroduplex mobility assay, which correlates very well with sequence analyses [24].

**Figure 1:**
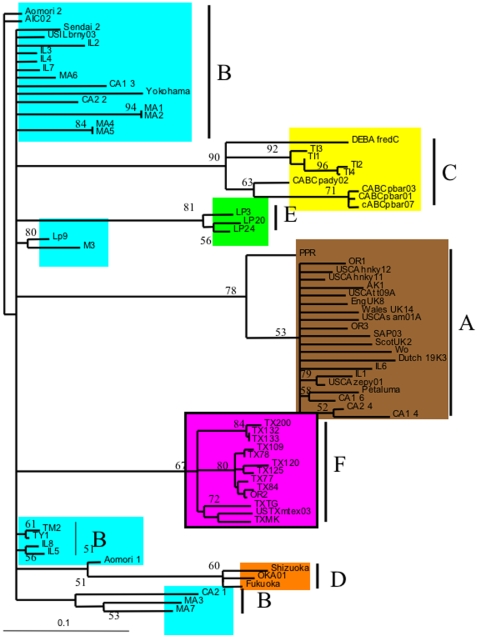
Rooted-quartet puzzling tree of the FIV envelope V3–V4 nucleotide sequence alignment. Support for the internal branches of the rooted tree topology is shown in percent of 100 puzzling steps.

**Figure 2:**
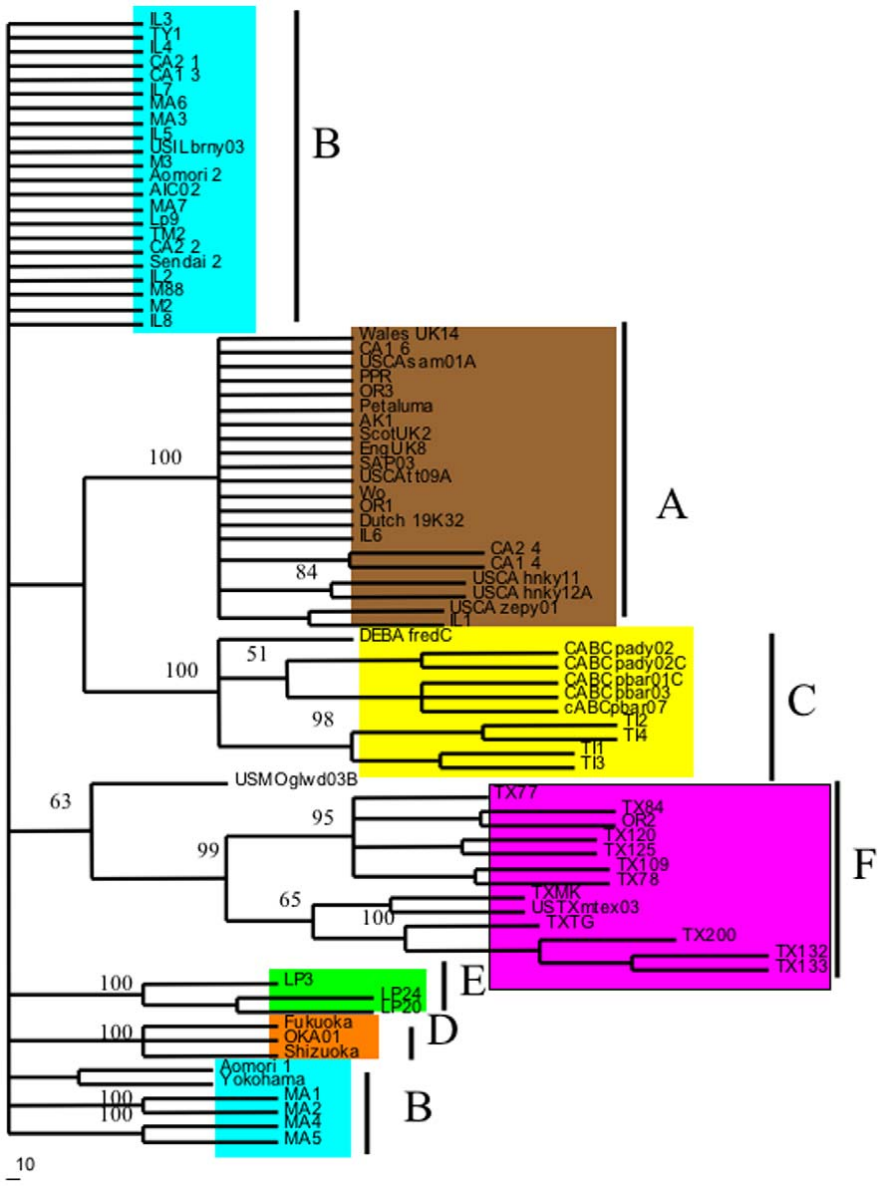
Maximum parsimony tree based on the nucleotide sequence of the V3–V4 envelope region. Values represent statistical bootstrap analysis based on 500 replicates. The tree was created using tree-bissection-reconnection and polytomies were created if maximum branch length is zero.

**Figure 3:**
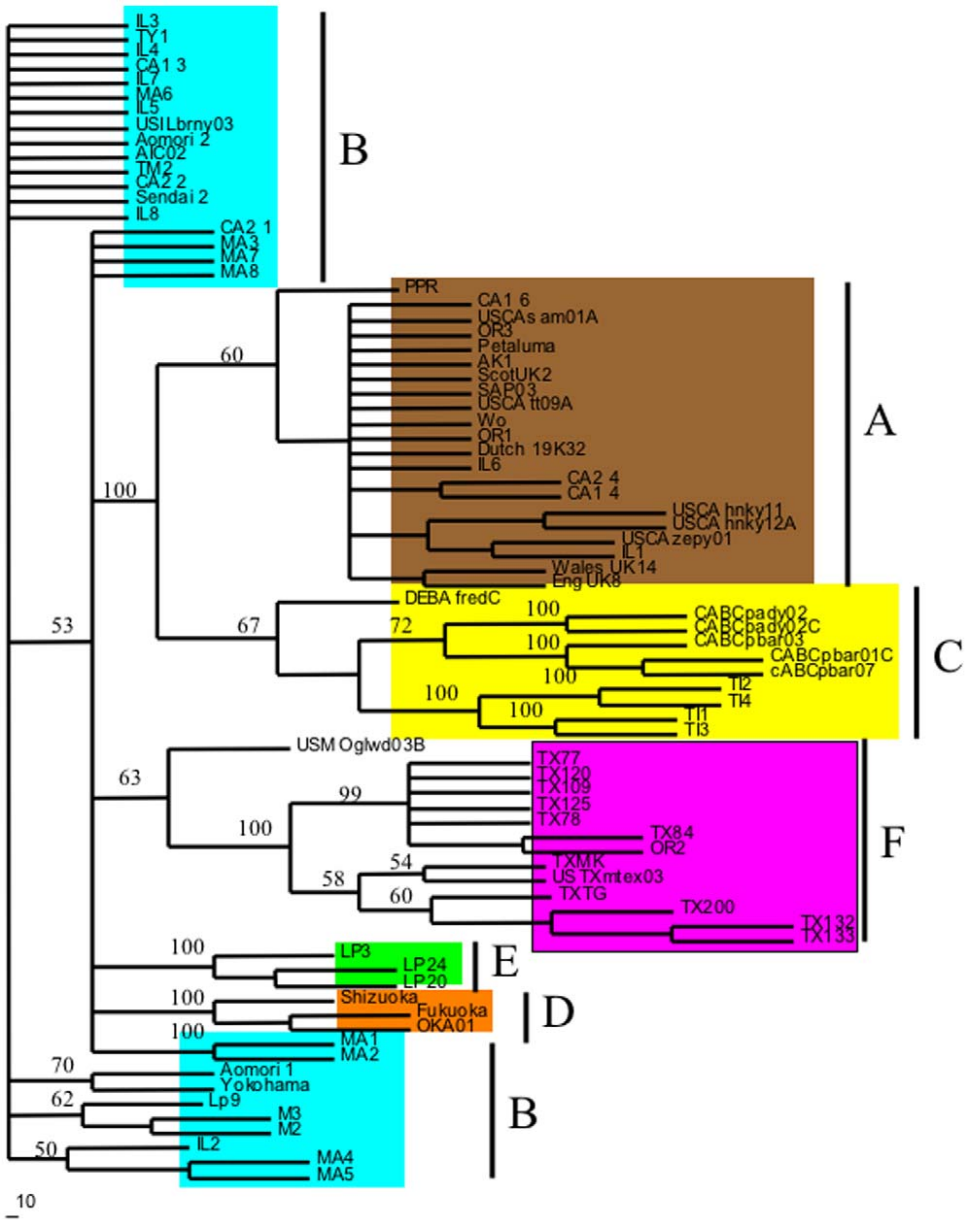
Log/Det paralinear distance tree based on the predicted amino acid sequence of the V3–V4 envelope region. Values represent statistical bootstrap analyses with full heuristic search at 1000 replicates. Tree-bissection-reconnection algorithm was used and zero branch lengths were not collapsed. All ties were broken at random.

A rooted-quartet-puzzling tree was used to analyze the sequence variation and clustering of the viral isolates as compared to sequences of previously published isolates. Tree puzzling uses maximum likelihood parameters and 100 puzzling steps were used to estimate statistical probabilities (Fig. 1). Polytomies were created if statistical probabilities were below 50%. As can be seen in figure 1 all of the TX isolates clustered to a unique and independent clade F. Also of interest, is that the previously published sequence, USTXmtex03, clustered to the very same clade, as well as an isolate from Oregon, OR2. Figure 1 also shows that many of the Oregon, California, and Illinois samples grouped to the A clade. Isolates IL2, IL3, IL4, IL5. IL7, IL8, MA1, MA2, MA3, MA4, MA5, MA6, MA7, CA1-2, CA2-2, and CA2-1 grouped to the B clade. This very same pattern was supported by the least stringent of all analyses, maximum parsimony and is illustrated in figure 2. Statistical support for maximum parsimony was obtained by 500 bootstrap replicates. Statistical analysis supported the unique F clade, as well as the clustering of the remaining isolates to the A and B clades (Fig. 2). Finally, using sequence data, a Log/Det paralinear distance tree was created (Fig. 3). The statistical values represent 1000 bootstrap replicates with full heuristic search (Fig. 3). This data was in complete agreement with the previous two methods of phylogenetic analysis used here and shows strong statistical support for the grouping of the isolates into clades (Fig. 3).

**Figure 4:**
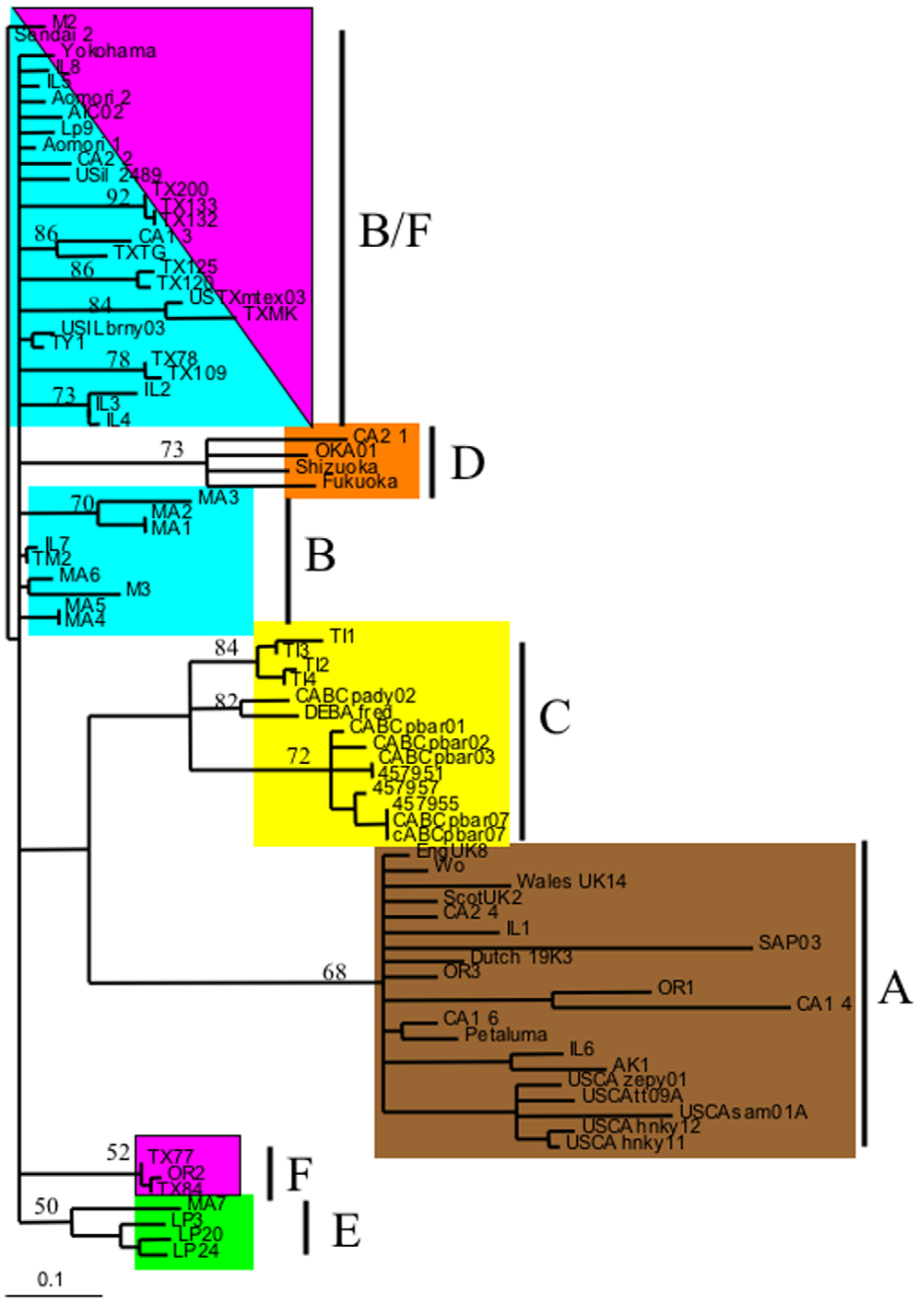
Quartet-puzzling tree based on approximate maximum likelihood values for the predicted envelope V3–V4 amino acid sequences. Bootstrap analysis was based on 1000 puzzling steps and values less than 50 were collapsed creating polytomies.

**Figure 5:**
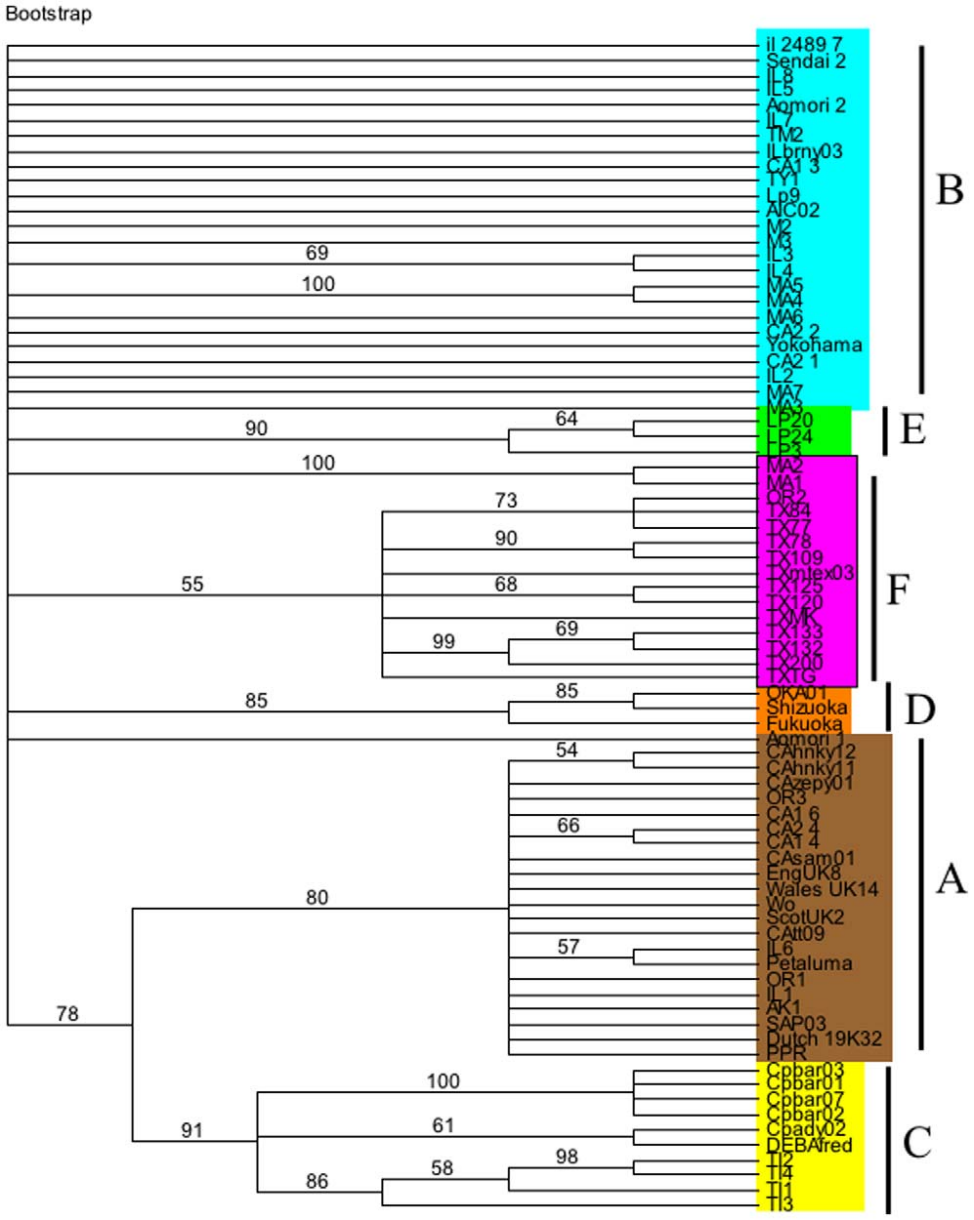
Maximum parsimony tree based on the predicted amino acid sequence of the V3–V4 envelope region. Values represent statistical bootstrap analysis based on 500 replicates. The tree was created using tree-bissection-reconnection and polytomies were created if maximumbranch length is zero.

Phylogenetic analyses were also performed using amino acid alignments. Figure 4 shows a quartet-puzzling tree based on the predicted amino acid sequence alignment. Statistical values were based on 1000 puzzling steps. Values less than 50 resulted in the collapse of the branch resulting in polytomies. Due to the complexity inherent to phylogenetic analysis based on amino acid alignments, many of the branches formed using nucleotide sequence data were collapsed (Fig. 4). Also, several of the isolates were not in agreement with previous analyses. For example, clades B, E and F were collapsed creating one large branch (Fig. 4). Isolate CA2-1, which had been previously grouped with the B clade, was grouped with the D clade (Fig. 4). However, when the amino acid alignments were subjected to analysis by maximum parsimony, the resulting tree was in agreement with the earlier nucleotide sequence data (Fig. 5). Again, the F clade contained the isolates OR2 and USTXmtex03 (fig. 5).

**Figure 6:**
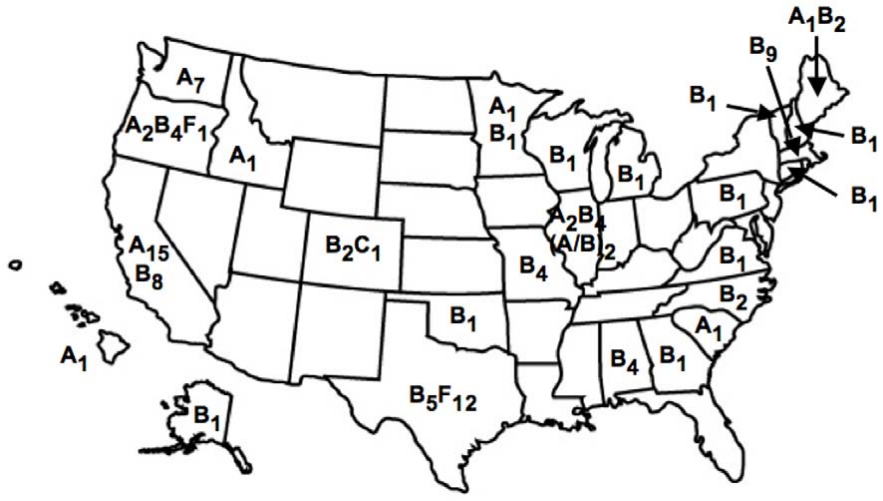
The clades of FIV isolates from various regions across the U.S.A. Data was obtained directly from this study as well as previously published studies. Clades and the number of viral isolates analyzed are shown as subscript values.

Figure 6 represents the geographic location and clade of the viral isolates, as well as the number of samples analyzed. Eleven states had clade A viral isolates, 20 states had clade B isolates, and only one state had clade C isolates (Fig. 6). Many of the states, such as Texas, Oregon, California, Illinois, and Minnesota had multiple clades (Fig. 6). Also, isolates from Oregon localized to 3 different clades and indicate a high level of viral heterogeneity. Table 1 shows the average percent nucleotide identity of the viral isolates as compared to previously published and characterized viral isolates (Table 1). The isolates, which grouped to the A and B clades by phylogenetic analysis, show levels of sequence divergence within the limitations of classification, 15.0%. There is one exception, isolate CA2-1 does not fall into the limits of classification to the B clade, yet is grouped with the B clade in 4 of 5 phylogenetic analyses (Table 1). Most of the isolates which group to the F clade do not fall within the limits for the previously classified clades A–E and represents the recently established clade F (Table 1) [35].

**Table 1:** The Average percent nucleotide identity of the viral isolates as compared to previously published and characterized viral isolates.

	Clade				
Isolate	A	B	C	D	E
A AK1	8.23	24.93	28.37	23.10	25.75
B CA1 3	25.39	9.99	24.66	20.57	20.37
A CA1 4	11.75	27.39	28.25	26.17	28.63
A CA1 6	6.88	24.01	25.04	21.72	23.15
B CA2 1	24.76	18.12	24.26	20.72	19.48
B CA2 2	24.21	7.95	20.71	18.71	17.23
A CA2 4	7.65	23.63	24.95	22.61	24.16
B IL1	8.75	27.94	27.40	26.29	26.32
B IL2	23.57	9.28	22.90	19.87	21.75
B IL3	22.79	5.31	21.73	18.50	17.03
B IL4	24.43	5.62	22.98	20.23	19.04
B IL5	23.00	5.19	22.70	19.65	17.75
B IL6	9.25	22.48	25.22	21.55	24.23
B IL7	25.29	5.79	23.43	20.56	19.29
B IL8	24.37	5.18	22.69	19.28	17.74
B MA1	26.51	12.34	23.28	20.40	17.02
B MA2	26.51	12.34	23.28	20.40	17.02
B MA3	25.59	14.13	23.35	18.78	18.87
B MA4	26.39	9.58	25.06	21.60	21.45
B MA5	26.39	9.58	25.06	21.60	21.45
B MA6	25.70	7.28	23.27	20.35	20.38
B MA7	24.39	14.63	21.31	19.52	15.29
A OR1	8.52	25.36	27.19	23.90	26.55
F OR2	25.58	15.40	24.86	22.78	19.96
A OR3	7.90	21.69	24.94	20.69	23.46
F TX77	24.28	14.49	24.00	22.04	18.47
F TX78	23.83	14.66	23.89	22.26	18.51
F TX84	25.15	15.78	25.27	23.00	19.89
F TX109	25.02	15.59	25.07	22.81	19.42
F TX120	23.92	14.90	23.75	21.78	18.23
F TX125	24.33	15.23	24.49	22.53	19.15
F TX132	25.13	16.68	24.73	23.21	17.08
F TX133	25.13	16.68	24.73	23.21	17.08
F TX200	24.82	17.21	25.08	23.68	17.09
F TXMK	26.26	16.33	25.63	24.49	19.73
F TXTG	26.78	16.43	27.42	23.40	20.10
F TXmtex	24.22	15.21	23.92	21.08	18.10

## Discussion

A major obstacle in generating a vaccine effective against FIV infection is the already large, and growing, genetic diversity among viral antigenic determinants, especially in regard to the area responsible for viral neutralization, the envelope. FIV vaccine researchers hope to circumvent this daunting task and still establish immunity against natural infection. However, little is known about the absolute sequence divergence of FIV. This limits researchers in their ability to develop and test vaccines capable of protection against natural challenge. The heteroduplex mobility assay (HMA) was developed as a rapid and reliable method for classifying HIV-1 env genes into clades and for inferring intraclade diversity. However, there are well over 200,000 HIV-1 sequences available through the National Center for Biotechnology Information, while only 646 sequences for FIV are available. Since the phylogenetic variation of HIV-1 has been well elucidated, HMA may be an appropriate means of determining genetic variation within a geographic region. However, in the case of FIV, much more sequence information is necessary to fully understand the similar diversity of FIV and HIV.

The first FIV infectious clones were derived from California isolates that represent clade A [37], [38]. Therefore, due to the advantages of using an infectious clone over a field isolate or a cell culture adapted isolate, many of the current vaccine studies are based on protection against a FIV clade A infection. However, the data from this study indicate that FIV sequence divergence may be greater than previously shown [11], [24], [31]. It is interesting to note that the majority of the isolates sequenced on the west coast, 24 of 34, were of clade A; whereas, the east coast isolates are, with the exception of one isolate, all of clade B (Fig. 6).

As indicated in a previous study, the B clade is much more difficult to define and is sometimes composed of sequences that do not fall into D or E clades [24]. The B clade had been previously broken into three clusters extending from a central stem. However, with the addition of the Texas isolates, the B clade is broken into two clearly definable and unique clades, the B clade and the TX clade. These TX isolates are on average greater than 15% divergent from the nearest clade, clade B. Due to the intense scope of the B clade, the TX clade has been established as a new viral clade, clade F [35].

Phylogenetic analyses and distance calculations presented in this study indicate that the viral divergence of FIV within the U.S.A. localize to 4 clades. Clades A and B are found throughout the U.S.A. and may represent the predecessor viruses. It is possible, that through isolation and genetic drift, the F clade may have been derived from one of the predecessor isolates. It is also possible that the F clade could represent a unique ancestral isolate itself, which was established from a geographically distinct region. The appearance of clade F in Oregon, as well as the clade C isolate in Colorado, may represent introduced, rather than endogenous, virus. The FIV isolates used in this study were from both domestic and feral cats. One possibility for these observations may be that these isolates were obtained from domestic cats that had been relocated by their owners. Relocation of a domestic FIV infected cat from the southwest to the northwest would explain the clade F in Oregon. Since clade C is typically found in Canada, the relocation of a domestic FIV infected cat from Canada to Colorado would explain the single clade C isolate. It is this conclusion that lead researchers to believe that a clade A virus had been introduced into the Japanese feline population from an American or European country [28].

Data presented from this study indicate that FIV may form 6 genetically distinct viral clades. This makes FIV a much more desirable model for HIV-1, which has been identified to have nine clades [39]. As there is a lack of biological and clinical analyses, there is no established correlation between clade and pathogenesis. To date, however, there are only 3 viruses, two clade A and one clade C, which have been shown to consistently cause fatal immunodeficiency disease in cats under experimental conditions and only one of these 3 has been molecularly cloned [38], [40], [41].

The development of an effective HIV vaccine for worldwide use may be modeled after FIV, which exemplifies the difficult challenges inherent to lentiviral vaccines. The challenge of high mutation rates, proviral DNA incorporation, viral reservoirs, and the multi-cellular tissue tropism of FIV make it the most suitable candidate model. Recently, a dual clade vaccine, consisting of a clade A virus and a clade D virus, was developed. However, this dual clade vaccine, which has been made commercially available in the U.S.A. only represents one of the 4 possible clades and provides limited immunity against cross clade challenge [34]. These phylogenetic studies may provide the tools necessary for identifying candidate strains for vaccine development. This study indicates that the current vaccine trials are negating the viral divergence of FIV and the inclusion of the 4 viral clades may be essential for creating a truly cross clade FIV vaccine capable of protecting cats within the U.S.A. from natural challenge.

## Supporting Information

Table S1 The FIV viral isolates, Genbank accession numbers and the origin of the env sequences used for phylogenetic analyses in this study.
(0.09 MB DOC)

Table S2 Sample descriptions and subtype analysis. a Subtype was determined by heteroduplex mobility assay [24]. b Subtype was determined based on the nucleotide sequence of the V3-V5 envelope region [31]. c Subtype was determined based on the nucleotide sequence of the V3-V4 envelope region as determined in this study.
(0.15 MB DOC)
