# Supplementary material for: A Detailed Phylogenetic Analysis of FIV in the United States
Source: PLoS One. 2010 Aug 9;5(8):e12004. doi: 10.1371/journal.pone.0012004 (PMC2918497; doi:10.1371/journal.pone.0012004)
Supplement: Table S1 — The FIV viral isolates, Genbank accession numbers and the origin of the env sequences used for phylogenetic analyses in this study. (0.09 MB DOC) [file pone.0012004.s001.doc]

Table S1. The FIV viral isolates, Genbank accession numbers and the origin of the env sequences usd for phylogenetic analyses in this study are shown below.

| **Name** | **Accession #** | **Origin** | **Name** | **Accession #** | **Origin** |
| --- | --- | --- | --- | --- | --- |
| AICO2 | AB010397 | Japan | MA7 | AF542138 | Massachusetts, USA |
| AK1 | AF542149 | Alaska, USA | OKA01D | AB010400 | Japan |
| Aomori 1 | D37816 | Japan | OR1 | AF542152 | Oregon, USA |
| Aomori 2 | D37817 | Japan | OR2 | AF542150 | Oregon, USA |
| CA1-3 | AF542154 | California, USA | OR3 | AF542151 | Oregon, USA |
| CA1-4 | AF542153 | California, USA | Petaluma | M25381 | California, USA |
| CA1-6 | AF542155 | California, USA | PPR | M36968 | California, USA |
| CA2-1 | AF542156 | California, USA | SAP03 | AB010404 | Japan |
| CA2-2 | AF542136 | California, USA | ScotUK2 | X69494 | United Kingdom |
| CA2-4 | AF542137 | California, USA | Sendai2 | D37814 | Japan |
| CABCpady02C | U02392 | Vancouver, BC | Shizuoka | D37811 | Japan |
| CABCpbar01C | U02393 | Vancouver, BC | TI1 | AB016025 | Taiwan |
| CABCpbar02C | U02394 | Vancouver, BC | TI2 | AB016026 | Taiwan |
| CABCpbar03C | U02395 | Vancouver, BC | TI3 | AB016027 | Taiwan |
| CABCpbar07C | U02397 | Vancouver, BC | TI4 | AB016028 | Taiwan |
| DEBAfredC | U57020 | Washington, USA | TM2 | M59418 | Yokyo, Japan |
| Dutch19K32 | M73965 | Amsterdam | TX77 | AY139103 | Texas, USA |
| EngUK8 | X69496 | United Kingdom | TX78 | AY139102 | Texas, USA |
| Fukuoka | D37815 | Japan | TX84 | AY139100 | Texas, USA |
| IL1 | AF542142 | Illinois, USA | TX109 | AY139102 | Texas, USA |
| IL2 | AF542139 | Illinois, USA | TX120 | AY139095 | Texas, USA |
| IL3 | AF542140 | Illinois, USA | TX125 | AY139094 | Texas, USA |
| IL4 | AF542141 | Illinois, USA | TX132 | AY139099 | Texas, USA |
| IL5 | AF542157 | Illinois, USA | TX133 | AY139098 | Texas, USA |
| IL6 | AF542158 | Illinois, USA | TX200 | AY139096 | Texas, USA |
| IL7 | AF542159 | Illinois, USA | TXMK | AY139097 | Texas, USA |
| IL8 | AF542160 | Illinois, USA | TXTG | AY139101 | Texas, USA |
| Lp3 | D84496 | Argentina | TY1 | D67064 | Japan |
| Lp9 | D84497 | Argentina | USCAhnky12A | U02403 | California, USA |
| Lp20 | D84498 | Argentina | USCAtt09A | U02413 | California, USA |
| Lp24 | D84500 | Argentina | USCAsam01A | U02410 | California, USA |
| M2 | X69501 | Pisa, Italy | USCAzepy01A | U02417 | California, USA |
| M3 | X69502 | Pisa, Italy | Usil2489_7B | U11820 | Illinois, USA |
| M88 | Y13868 | Pisa, Italy | USILbrny03B | U02418 | Illinois, USA |
| MA1 | AF542143 | Massachusetts, USA | USMAboy03B | U02419 | Massachusetts, USA |
| MA2 | AF542144 | Massachusetts, USA | USMOglwd03B | U02420 | Missouri, USA |
| MA3 | 542145 | Massachusetts, USA | USTXmtex03B | U02422 | Texas, USA |
| MA4 | 542146 | Massachusetts, USA | Wales UK14 | X69497 | Colymyn Bay, Wales |
| MA5 | AF542149 | Massachusetts, USA | Wo | L06312 | France |
| MA6 | AF54214 | Massachusetts, USA | Yokohsma | D37812 | Japan |
