# Supplementary material for: A Detailed Phylogenetic Analysis of FIV in the United States
Source: PLoS One. 2010 Aug 9;5(8):e12004. doi: 10.1371/journal.pone.0012004 (PMC2918497; doi:10.1371/journal.pone.0012004)
Supplement: Table S2 — Sample descriptions and subtype analysis. a Subtype was determined by heteroduplex mobility assay [24]. b Subtype was determined based on the nucleotide sequence of the V3-V5 envelope region [31]. c Subtype was determined based on the nucleotide sequence of the V3-V4 envelope region as determined in this study. (0.15 MB DOC) [file pone.0012004.s002.doc]

Table S2. Sample descriptions and subtype analysis.

| **Sample Code** | **State** | **Source** | **Clinical Symptoms** | **Clade** |
| --- | --- | --- | --- | --- |
| USAStink | AL | Stevens | Skin lesion, Conjunctivitis | B^a^ |
| USALtom | AL | Stevens | Fight Abscess, Lymphadenopathy | B^a^ |
| USALsylv | AL | Steele | Peridontal disease | B^a^ |
| USALspuz | AL | Steele | Sneezing, fight abscess, fever, depression | B^a^ |
| AK1 | AK | Sherwood | Chronic proliferative otitis exreena, cocci and aural haematoma, fight abscesses | A^c^ |
| USCAbogi | CA | Kitchell | Diarhea, glaucoma | A^a^ |
| USCAlucy | CA | Suranyi | Moderately severe gingivitis | A^a^ |
| USCAmrbl | CA | Hutson | Wt. loss, gingivitis | A^a^ |
| USCApine | CA | L. Wengren | N/A | A^a^ |
| USCAsoph | CA | Kitchell | Pernicous anemia, cholangiohepatitis | A^a^ |
| USCAspph | CA | Kitchell | Asymptomatic | A^a^ |
| USCAstan | CA | Benjamin | Asymptomatic | A^a^ |
| USCAbud | CA | Hutson | Conjunctivitis, salivary gland tumor, wt. Loss | B^a^ |
| USCArust | CA | Stevens | Fight abscess, lymphadenopathy | B^a^ |
| USCAsams | CA | Stevens | Wt. loss, UTI, dental disease | B^a^ |
| USCAtant | CA | Stewart | Lymphadenopathy | B^a^ |
| USCAalem | CA | N/A | Wt. loss, opportunistic infections | A^b^ |
| USCAhnky | CA | N/A | Wt. loss, opportunistic infections, diarrhea | A^b^ |
| USCAatt | CA | N/A | Wt. loss, abnormal motor behavior | A^b^ |
| USCAzepy | CA | N/A | Asymptomatic | A^b^ |
| USCAsam | CA | N/A | Abnormal motor behavior | A^b^ |
| USCAherr | CA | L. Wengren | N/A | B^b^ |
| CA1-3 | CA | B. Sanderson | Severe gingivitis | B^c^ |
| CA1-4 | CA | B. Sanderson | Asymptomatic | A^c^ |
| CA1-6 | CA | B. Sanderson | Asymptomatic | A^c^ |
| CA2-1 | CA | T. Noyes | Benign aprocrine cysts | B^c^ |
| CA2-2 | CA | T. Noyes | Asymptomatic | B^c^ |
| CA2-4 | CA | T. Noyes | Asymptomatic | A^c^ |
| USCOtoby | CO | O’Neil | Diarrhea, gingivitis | B^a^ |
| USCOscru | CO | Ediger | Wt. loss | C^a^ |
| USCOgrou | CO | Batch | Asymptomatic | B^a^ |
| USCTgrif | CT | Mullin | Wt. loss, depression, weakness, bite wounds | B^a^ |
| USHIsam | HI | McBane | Renal failure, cysts, heartworm | A^b^ |
| USIDjake | ID | Norman | Wt. loss, gingivitis | A^b^ |
| USILbb_1 | IL | Houghton | Fever, wt. loss, dental absseces | A/B^a^ |
| USILbrny | IL | N/A | Asymptomatic | Bb |
| USILbb_2 | IL | Houghton | Fever, wt. loss, dental absseces | A/B^a^ |
| USILjoey | IL | Nordwall | Ginigivitis | B^a^ |
| USILsque | IL | Currigan | N/A | B^a^ |
| IL1 | IL | D. Khula | Fight absseces, conjunctivitis | A^c^ |
| IL2 | IL | D. Khula | Chronic upper respiratoy infection, diabetic | B^c^ |
| IL3 | IL | D. Khula | Chronic otitis | B^c^ |
| IL4 | IL | D. Khula | Dental disease, ringworms | B^c^ |
| IL5 | IL | D. Khula | N/A | B^c^ |
| IL6 | IL | D. Khula | N/A | A^c^ |
| IL7 | IL | D. Khula | N/A | B^c^ |
| IL8 | IL | D. Khula | N/A | B^c^ |
| USINsamy | IN | Albate | Dehydration | A^a^ |
| USLAgeor | LA | Bohm | Asymptomatic | B^a^ |
| USMEmowg | ME | Hoyt | Asymptomatic | A^a^ |
| USMEjack | ME | Hoyt | Facial abscesses | B^a^ |
| USMEchar | ME | Beekman | Asymptomatic | B^a^ |
| USMDsilv | MD | Kriemelmeyer | Ulceration, stomatitis | B^a^ |
| USMDeliz | MD | Brunt | Lymphadenopathy, dermatitis | B^a^ |
| USMDsidn | MD | Brunt | Vomiting | B^a^ |
| USMAsboy | MA | N/A | Asymptomatic | B^b^ |
| USMAkitt | MA | Ricksgers | Dental disease | B^a^ |
| MA1 | MA | Penny | Dental disease | B^c^ |
| MA2 | MA | Penny | Dental disease | B^c^ |
| MA3 | MA | Penny | Fight abscesses, dental disease | B^c^ |
| MA4 | MA | Penny | Dental disease | B^c^ |
| MA5 | MA | Penny | Dental disease, fight abscesses, upper respiratory infection, sinus arthymia | B^c^ |
| MA6 | MA | Penny | Wt. loss, dental disease | B^c^ |
| MA7 | MA | Penny | Wt. loss, dental disease, fight wounds | B^c^ |
| USMImrle | MI | Houston | Asymptomatic | B^b^ |
| USMNbust | MN | U. Minnesota | Acute renal failure | A^a^ |
| USMNnewt | MN | DeJong | Asymptomatic | B^a^ |
| USMOmrki | MO | Sundahl | Dental disease | B^a^ |
| USMOpook | MO | Lloyd | Sinusitis | B^a^ |
| USMOglwd | MO | N/A | Asymptomatic | B^b^ |
| USMOrasc | MO | Lloyd | Diarrhea, gingivitis | B^a^ |
| USNHluca | NH | Sobel | Upper respiratory infection | B^a^ |
| USNCboom | NC | Kuhn | Asymptomatic | B^a^ |
| USNCramb | NC | Bbissel | Gingivitis | B^a^ |
| USOKlgrl | OK | N/A | Asymptomatic | Bb |
| OR1 | OR | L. Kennon | Dental disease, dermatitis | A^c^ |
| OR2 | OR | L. Kennon | Fight Abscesses, dental disease, dermitits | TX^c^ |
| OR3 | OR | L. Kennon | Fight Abscesses, dental disease, dermitits | A^c^ |
| USPAgeor | PA | Wooley | Asymptomatic | B^a^ |
| USSCboge | SC | McCullers | Asymptomatic | A^a^ |
| USTXkizz | TX | McManis | Asymptomatic | B^a^ |
| USTXoliv | TX | McManis | Asymptomatic | B^a^ |
| USTXtigg | TX | McCaine | Gingivitis, stomatitis | B^a^ |
| USTXyefe | TX | McCaine | Gingivitis, stomatits | B^a^ |
| USTXmtex | TX | N/A | Asymptomatic | Bb |
| TX77 | TX | Bernstein/Slater | Fight Abscesses, ovarian cyst, roundworms | F^c^ |
| TX78 | TX | Bernstein/Slater | Roundworms, tapeworms | F^c^ |
| TX84 | TX | Bernstein/Slater | Asymptomatic | F^c^ |
| TX109 | TX | Bernstein/Slater | Asymptomatic | F^c^ |
| TX120 | TX | Bernstein/Slater | Lymphoid hyperplasia | F^c^ |
| TX125 | TX | Bernstein/Slater | Chronic dermatitis, roundworms, lymphoid hyperplasia | F^c^ |
| TX132 | TX | Bernstein/Slater | Roundworms | F^c^ |
| TX133 | TX | Bernstein/Slater | N/A | F^c^ |
| TX200 | TX | Bernstein/Slater | Lymphoid hyperplasia, hypertrophic cardiomyopathy | F^c^ |
| TXMK | TX | Bernstein/Slater | Skin lesions, fight wounds | F^c^ |
| TXTG | TX | Bernstein/Slater | Skin lesions, fight wounds | F^c^ |
| USVIcarm | VI | Quatmann | Depression, wt. loss | B^a^ |
| USWAabk1 | WA | J. Abkovitz | N/A | A^a^ |
| USWAabk3 | WA | J. Abkovitz | N/A | A^a^ |
| USWAabk4 | WA | J. Abkovitz | N/A | A^a^ |
| USWAabk5 | WA | J. Abkovitz | N/A | A^a^ |
| USWAflee | WA | Faler/Fellows | Abscess, lymphadenopathy, respiratory infection | A^a^ |
| USWAkikl | WA | Faler/Fellows | Dental disease, wt. loss | A^a^ |
| USWAsea7 | WA | C. Grant | N/A | A^a^ |
| USWItaz | WI | Helfand | Gingivitis, stomatitis | B^a^ |
